# Malnutrition in Chronic Pancreatitis: Causes, Assessment Methods, and Therapeutic Management

**DOI:** 10.1155/2020/8875487

**Published:** 2020-08-08

**Authors:** Agnieszka Madro

**Affiliations:** Medical University, Department of Gastroenterology with Endoscopic Unit, Lublin, Poland

## Abstract

**Purpose:**

In recent years, more and more emphasis has been placed on early diagnosis and adequate treatment of malnutrition in the course of chronic diseases (CP). One of these diseases is chronic pancreatitis in which malnutrition may develop as a consequence of abdominal pain, vomiting, diarrhea, and alcohol abuse. The aim of this review paper is recognized if we can improve the nutritional status of patients with CP.

**Methods:**

This paper is based on systematic literature review according to the PubMed.

**Results:**

One of the most important problems is lack of “gold standard” in screening of nutritional status in patients with CP, especially in outpatient clinics. Another problem is preventing malnutrition in these patients and beginning treatment already at significant stages of disease. To prevent malnutrition you must first recognize the causes of malnutrition in CP, adequately assess its severity using one of available questionnaires and then apply the appropriate therapeutic management. At each visit, remember to assess the nutritional status of the patient, including laboratory markers and anthropometric measurements. Patients should be advised to stop smoking and drinking alcohol and to use adequate enzyme supplementation.

**Conclusion:**

Patients with CP should be led by a team of gastroenterologist, diabetologist, and psychologist and consulted by a dietitian, specialist of pain treatment, and surgeon.

## 1. Introduction

In recent years, more and more emphasis has been placed on early diagnosis and adequate treatment of malnutrition in the course of chronic diseases. Its occurrence is associated with a higher rate of hospitalization, an increased risk of complications, and the costs of treatment and it causes higher mortality [1, 2].

Chronic pancreatitis (CP) is a chronic inflammatory process of the pancreas caused by recurrent inflammatory episodes characterised by irreversible morphological changes with replacement of the pancreatic parenchyma by fibrous connective tissue. The result of morphological changes is progressive exocrine and endocrine insufficiency of the pancreas. The consequences of these phenomena are clinical symptoms: pain, malnutrition, and diabetes mellitus. Malnutrition is common among patients with CP and may develop as a result of abdominal pain, vomiting, diarrhea, and alcohol abuse [3]. Diabetes mellitus may potentiate this phenomenon [4].

One of the most important problems is lack of “gold standard” in screening of nutritional status in patients with CP, especially in outpatient clinics. There are several scales used to screen patients in hospitals: nutritional risk screening (NRS-2002), malnutrition screening tool (MST), and short nutritional assessment questionnaire (SNAQ) [5]. In most patients with CP, nutritional status is assessed only on body weight and basic biochemical blood parameters. In clinical trials, the CONUT procedure is used, based on measuring albumin, total lymphocyte count, and cholesterol [6]. Malnutrition becomes a great problem in patients with CP, which is underestimated by doctors and nurses. Little attention is paid to preventing malnutrition in these patients by beginning treatment early at significant stages of the disease. Accurate statistics on malnutrition in CP are not known, and many doctors consider this problem as insignificant [7]. The aim of this review paper is recognized if we can improve the nutritional status of patients with CP.

## 2. Methods

This paper is based on systematic literature review according to the PubMed.

### 2.1. Causes of Malnutrition in CP

Malnutrition in CP has several causes that can overlap. We can divide them into three main groups depending on the disease, on the patient, and on the doctor [8].

The essence of the disease is the progressive reduction of pancreatic exocrine function, which results in reduced production of pancreatic enzymes and thus impaired digestion of all major nutrients [9].

Triglyceride digestion is particularly disturbed, resulting in reduced absorption of fats and fat-soluble vitamins such as A, D, E, and K, and impaired secretion of proteases can cause protein malnutrition and vitamin B12 deficiency. In extreme, untreated cases, this leads to fatty diarrhea. One of the main symptoms of CP is pain after meals, which limits the patient's consumption. In turn, chronic inflammation can induce the anabolic state which impairs protein usage. Diabetes that appears in advanced CP is usually unstable, with high blood glucose fluctuations, which leads to appetite disorders [9]. The patient-dependent causes are primarily the use of stimulants: smoking and drinking alcohol despite the diagnosis of the disease. Such an action aggravates malnutrition. The impact of continuing alcohol consumption on the nutritional status should be discussed with the patients. In addition, patients abusing alcohol do not attend regular check-ups, do not use adequate enzymatic supplementation, and do not undergo systematic lab tests. The patients visiting the doctor play a major role in preventing malnutrition. Unfortunately, knowledge about CP is insufficient among general practitioners. Insufficient enzymatic supplementation is often used, too little time is devoted to patient education (time and patience). and no screening for malnutrition and diabetes is performed. Doctors do not use adequate supplementation of micronutrients and vitamins, do not properly treat pain, and refer to surgery too late. Also, proper attention is not paid to the regular consultation of dietetics specialists, and the advices given by doctors or nurses are often insufficient [10].

### 2.2. Malnutrition Assessment

As I mentioned above, there is no gold standard in assessing the nutritional status of patients with CP, especially in outpatient care. Body mass index (BMI) assessment is insufficient, even in combination with albumin and prealbumin evaluation [11]. However, albumin is an acute phase protein, its reduced concentration is also observed in infections, burns, overhydration, liver failure, cancers, and nephrotic syndrome, so it is not a reliable marker of malnutrition. In addition, albumin concentration has been observed to remain unchanged until advanced fasting (BMI <12 or fasting period >6 weeks) [11].

Therefore, albumin cannot be a reliable marker for diagnosing protein-energy malnutrition [12]. A more reliable marker of nutritional assessment is prealbumin, its half-life is much shorter (2-3 days), and its total pool is smaller. However, its concentration may also be reduced in infections, hyperthyroidism, and liver failure and elevated in the case of renal failure and hypothyroidism [13]. Transferrin is another plasma protein suggested to be useful in assessing malnutrition, but its concentration depends on iron metabolism and renal function [14]. In a study conducted in patients with different body weights, including malnourished ones, no correlation between fat free mass and transferrin concentration was observed [15]. Retinol-binding protein (RBP) is often lower in CP patients than in healthy individuals and has frequently been considered as a sign of protein-calorie malnutrition [16]. However, all of the above biochemical markers, except albumin, are not routinely determined in clinical practice, especially in outpatient care. Due to recent reports emphasizing the role of the inflammatory process in the pathogenesis of malnutrition, attention has been paid to the C-reactive protein (CRP). Although its concentration is influenced by factors not related to malnutrition such as inflammatory processes, it helps to differentiate whether the concentration of total protein decreases due to the presence of inflammation or due to insufficient supply as in the case of malnutrition [17].

Faecal elastase -1 is a simple tool for early diagnosis of pancreatic exocrine insufficiency (PEI). There are no other simple tools similar to faecal elastase-1 for early diagnosis of pancreatic exocrine insufficiency (PEI). Faecal elastase-1 is the most sensitive early test, and imaging examinations are the tests of choice for the detection of chronic pancreatitis. Despite the increasing availability of this test, it still does not function in the general understanding among GPs and is not being used. Every patient with newly diagnosed CP should be screened for PEI with faecal elastase-1. This allows for early implementation of adequate enzymatic supplementation, which can prevent malnutrition [18, 19].

The assessment of malnutrition may be helpful measurements of anthropometric parameters such as body mass index (BMI), determination of triceps skin fold (TSF), midarm circumference (MAC), determination of handgrip strength (HSG), and bioelectrical impedance analysis. In practice, apart from anthropometric measurements, other tests are not widely used, as they require the use of specialized equipment that is not widely available [20].

Recently, many studies have been published whose purpose was to estimate malnutrition in patients with CP. Greer et al. evaluated 301 patients with CP and 266 from the control group for malnutrition. Attention was paid to significantly lower BMI than in the control group; however, as much as 44.5% of patients with CP were overweight or obese. No significant differences in BMI were noted in people suffered from CP for less than 5 years. However, statistically significant lower levels of vit. A and E, osteocalcin, and prealbumin retinol-binding protein in patients with CP were noted, but this difference was not shown for vit. D. In turn, unexpectedly statistically significant higher values of vit. B12 in patients with CP were observed. The authors of the paper cannot explain this phenomenon, because in relation with the deficiency of protease one should expect lower values of B12 vitamins in the serum [9]. Researchers confirmed earlier reports [21] that CRP is slightly but statistically significantly higher in patients with CP, while the level of TNF-alpha was in most patients with CP below the detection threshold. Important conclusions of the work are that the presence of diabetes and drinking alcohol did not affect the biomarkers of nutrition and inflammation, but had an impact on the state of nutrition. In turn, a negative effect of smoking on biomarkers of nutrition and inflammation was observed. Systematic use of enzymatic and vitamin supplementation improves the nutritional status of patients with CP regardless of the etiology [9].

An important problem that arises in the course of CP is the aggravation of malnutrition as the disease progresses. Hintaran et al. [22] studied the changes in the nutritional status of patients with CP together with the duration of the disease and the impact of performing systematic assessment on the nutritional status in the Dutch population. The study involved 50 people with CP who were assessed at the starting point and after 2 years. Each person completed the SF-36 questionnaire that covers several domains of health perception. Nutritional assessment consisted of the mini nutritional assessment (MNA), antropometric measurement, determination of handgrip strength (HGS), bioelectrical impedance analysis (BIA), and faecal and serological biomarkers [22]. Twenty-eight patients underwent a reevaluation of the nutritional status after approx. 2 years. There were no significant differences in the quality of life after this period of illness. The authors emphasize that in assessing nutritional status it is more important to assess body composition than body weight. The researches found statistically significant increases in waist circumference, hip circumference, upper arm muscle area, midarm muscle circumference, fat mass, and fat mass index. Free fat mass assessment, which also correlates with a decrease in muscle strength, seems particularly important. On the contrary, statistically significant decreases in midarm circumference, triceps skin fold, handgrip strength, fat free mass, and fat free mass index have been observed. Additionally, handgrip strength assessment appears to be useful for estimation of any loss in fat free mass, being more indicative of malnutrition than weight loss [22].

### 2.3. Therapeutic Management

One of the most important issues in treating malnutrition in CP must be to normalize digestion through the adequate enzyme supplementation. It has been shown to improve weight, reduce faecal fat secretion, ameliorate abdominal pain, and improve quality of life. Such management does not show side effects. A minimum lipase dose of 40000–50000 Ph.U. is recommended with main meals and half that dose with snacks. The most effective are enteric-coated microspheres or mini-microspheres of <2 mm size. Micro- or mini-tablets of 2.2–2.5 mm size may be also effective [18, 23].

The response to enzyme substitution should be assessed not only on the basis of symptoms but also on the basis of normalization of plasma malnutrition markers such as fat-soluble vitamins, retinol-binding protein, albumin, prealbumin, minerals (iron, zinc, and magnesium). It is not recommended to reduce dietary fat in patients with CP; this procedure may result in lower secretion of endogenous enzymes. If there is no improvement in nutrition, turn on hydrochloric acid suppression, exclude small intestine bacterial overgrowth (SIBO), celiac disease, inflammatory bowel disease (IBD), lactose intolerance, and reduced bile acid absorption [23].

One of the most common complications of malnutrition in patients with CP is progressive osteoporosis. This phenomenon was not well understood, so a multicenter study was carried out to recruit patients from several countries [24]. Osteopenia was found in 40% of patients and osteoporosis in 25%, in all these patients the BMI was lower than that in the remaining patients with CP. The risk of osteoporosis was greater in older women with lower BMI. As many as 32% of patients had vitamin K deficiency, which significantly increased the risk of osteoporosis in men. Interestingly, vitamin D and PEI deficiency were not associated with an increased risk of osteopathy. An important conclusion from this work is the need to perform bone mineral density tests (densitometry), determination of vitamin D and K in serum, and in the case of deficiencies their simultaneous supplementation. An underestimated problem in patients with CP is sarcopenia. Meanwhile, recent studies have confirmed that sarcopenia is an independent risk factor for complications, increased hospitalization, and reduced survival [25]. Sarcopenia was measured by SMI (skeletal muscle mass index), HGS (handgrip strength), and TUG (timed up-and-go test) in relation to age and gender. Most patients with sarcopenia had normal body weight and BMI, or were obese. Sarcopenia has been associated with poorer quality of life, less physical activity, increased hospitalization, and shorter survival. Exocrine pancreatic insufficiency was an independent and significant risk factor for sarcopenia [25].

The role of intestinal microbiota in the course of CP and its effect on metabolic processes, including malnutrition, is also an important issue [26]. Patients with diabetes in the course of CP had a significantly longer disease duration, so they should be expected to have more disturbances in the intestinal microbiota [27].

This observation was confirmed in Sai Manasa Jandhyala's publication [28]. It was found that patients with diabetes in the course of CP are more malnourished. An increase in the *Firmicutes*:*Bacteroidetes* ratio is observed in patients with and without diabetes mellitus; reduction in the amount of *Faecalibacterium prausnitzii* (which is one of the most important commensals of the human intestine) in patients with CP and a negative correlation between this bacterium and circulating endotoxin is observed. Increased endotoxin synthesis was found in patients with CP and associated diabetes. Reducing the amount of *Ruminococcus bromii* strain in patients with CP impairs the mucosal barrier and metabolism, because this bacterium breaks down enzyme-resistant starch. Dysbiosis was dependent on the duration of the disease and its severity. Correlations between bacteria and selected clinical parameters were also assessed. Among others, a significant positive correlation of *Faecalibacterium prausnitzii* abundance with plasma insulin levels was found. However, there was no correlation of *Ruminococcus bromii* abundance with plasma endotoxin and blood glucose. The researches observed negative correlation of *Bacteroides* and *Escherichia* with BMI; negative correlation of *Akkermansia* with PEI; positive correlation of *Lactobacillus* with serum prealbumin; positive correlation of *Prevotella* with serum vitamin B12; negative correlation of *Clostridium* with serum vitamin B12; and positive correlation of *Shigella* with plasma endotoxin level. Changing bacterial microflora may be important in preventing or delaying the development of metabolic complications, including diabetes and thus preventing malnutrition [28]. In the most recent paper, Zhou at al. examined gut microbiota in patients with CP in comparison to the healthy control. They observed gut microbiota dysbiosis with decreased diversity and richness and taxa-composition changes. The gut microbiome of the CP group showed lower *Firmicutes* and *Actinobacteria* abundances than the control group and higher *Proteobacteria* abundances. The abundances of *Escherichia, Shigella,* and other genera were high in the CP group, whereas that of *Faecalibacterium* was low. The authors confirmed that patients with CP have gut microbiota dysbiosis that may be partly affected by pancreatic exocrine function [29].

An important issue related to dysbiosis is the relationship between small intestinal bacterial overgrowth (SIBO) and PEI in chronic pancreatitis. Capurso et al. reviewed data from nine publications and on this basis they concluded that one-third of CP patients have SIBO with a range between 14 and 92% and considerable heterogeneity. The prevalence of SIBO seems to depend on the type of test used for diagnosis and also on the inclusion criteria for CP patients, with higher positivity rates in studies including patients who previously underwent surgery. The relationship between the presence and severity of symptoms and the diagnosis of SIBO in CP patients seemed to vary across the different studies. A higher rate of symptoms in patients with SIBO was reported in only three of the five studies. The treatment of SIBO with antibiotics in CP patients may result in fewer symptoms [30].

Some of the patients with CP, despite compliance with the recommendations (stop smoking and drinking alcohol), use of adequate enzyme supplementation, and antipain treatment, still have complaints and insufficient nutrition. In this case, the patient should be immediately referred to a surgical consultation to consider surgical treatment. Such proceedings should not be postponed until cachexia of the patient is observed, and then the risk of performing the procedure is very high. Surgical management in CP consists of 2 strategies: pancreatic duct drainage and resection of the targeted lesion (pancreatectomy). The first concept includes pancreatojejunostomy with distal pancreatectomy, Puestow's procedure [31] and longitudinal pancreatojejunostomy (Partington's procedure) [32]. The second concept includes distal pancreatectomy (DP), pancreaticoduodenectomy (PD), and duodenum-preserving pancreatic head resection (Beger's procedure) [33]. The technique that consists of both drainage and resection is the Frey's procedure [34]. Sato H. recently published a paper assessing the usefulness of the Frey's procedure not only in reducing pain but also in improving nutritional status compared to pancreaticoduodenectomy. Nutritional status was assessed using the CONUT protocol (albumin, total lymphocyte count, and cholesterol). This study found that the postoperative nutritional status of CP patients improved significantly after Frey's procedure, whereas no nutritional changes were observed after PD. Authors suggest three main mechanisms involved in the nutritional status improvement: pain relief allowing for resumption of oral intake; reducing the chronic inflammation and removing the pancreatic duct obstruction and as a consequence improvement in pancreatic exocrine function; Frey's procedure preserves also the pancreatic parenchyma and maintains the potential source of pancreatic juice and insulin. Generally, surgery should not be delayed until the patient is severely cachectic, and the type of surgery should depend on the location of the inflammatory lesions, anatomical conditions, and the center's experience [32]. There are no data in the literature regarding the impact of endoscopic treatment on the improvement of nutrition in patients with chronic pancreatitis.

## 3. Main Recommendations

In 2018, recommendations from the United European Gastroenterology evidence-based guidelines for the diagnosis and therapy of chronic pancreatitis were published, some of which concern the prevention, diagnosis, and treatment of malnutrition. The recommended tool in screening is MUST (malnutrition universal screening tools) or NRS, anthropometric measurements (BMI, arm circumference, skin fold under the triceps), and hand muscle strength were found useful. It is also advisable to assess the deficiency of albumin, fat-soluble vitamins (K and D), and microelements Fe, Zn, and Mg supplementation should only be used in the case of deficiencies. The most important in treatment was adequate enzymatic substitution and eating small, frequent, high-energy meals without limiting dietary fat. Also, high-fiber products should be avoided. If you have difficulty in following a proper diet, consult your dietitian. In turn, enteral nutrition is indicated in patients who do not respond to oral nutrition, with delayed intestinal emptying, with pain and nausea and/or vomiting. Parenteral nutrition is only recommended for patients with gastrointestinal obstruction, fistulas, or deep malnutrition before elective surgery [18].

## 4. Summary

Patients with CP should be led by a team of gastroenterologist, diabetologist, and psychologist and consulted by a dietitian, specialist of pain treatment, and surgeon. At each visit, remember to assess the nutritional status of the patient, including laboratory markers and anthropometric measurements. Patients should be advised to stop smoking and drinking alcohol and to use adequate enzyme supplementation. It is necessary to treat pain and diabetes and to respond to the patient's worsening nutrition: consideration of enteral or parenteral nutrition and even surgery. Because of this procedure, we are able to improve the nutritional status of patients with chronic pancreatitis.

## Data Availability

All data contained in the manuscript are available in the PubMed database. The reader can find all the cited articles in this widely used database.
